# Combining accelerometry with allometry for estimating daily energy expenditure in joules when in-lab calibration is unavailable

**DOI:** 10.1186/s40462-023-00395-0

**Published:** 2023-05-30

**Authors:** Pritish Chakravarty, Gabriele Cozzi, David Michael Scantlebury, Arpat Ozgul, Kamiar Aminian

**Affiliations:** 1 Institute of Bioengineering, Ecole Polytechnique Fédérale de Lausanne, Lausanne, Switzerland; 2 Department of Evolutionary Biology and Environmental Studies, Universität Zürich, Zurich, Switzerland; 3 Department for the Ecology of Animal Societies, Max Planck Institute of Animal Behavior, Constance, Germany; 4 Kalahari Research Centre, Kuruman River Reserve, Van Zylsrus, 8467 South Africa; 5 School of Biological Sciences, Queen’s University Belfast, Belfast, Northern Ireland, UK

**Keywords:** Daily energy expenditure, Dynamic body acceleration (DBA), Allometry, Speed estimation, Kalman filter, Doubly labelled water, Accelerometer, Gyroscope

## Abstract

**Background:**

All behaviour requires energy, and measuring energy expenditure in standard units (joules) is key to linking behaviour to ecological processes. Animal-borne accelerometers are commonly used to infer proxies of energy expenditure, termed ‘dynamic body acceleration’ (DBA). However, converting acceleration proxies (m/s^2^) to standard units (watts) involves costly in-lab respirometry measurements, and there is a lack of viable substitutes for empirical calibration relationships when these are unavailable.

**Methods:**

We used past allometric work quantifying energy expenditure during resting and locomotion as a function of body mass to calibrate DBA. We used the resulting ‘power calibration equation’ to estimate daily energy expenditure (DEE) using two models: (1) locomotion data-based linear calibration applied to the waking period, and Kleiber’s law applied to the sleeping period (ACTIWAKE), and (2) locomotion and resting data-based linear calibration applied to the 24-h period (ACTIREST24). Since both models require locomotion speed information, we developed an algorithm to estimate speed from accelerometer, gyroscope, and behavioural annotation data. We applied these methods to estimate DEE in free-ranging meerkats (*Suricata suricatta*), and compared model estimates with published DEE measurements made using doubly labelled water (DLW) on the same meerkat population.

**Results:**

ACTIWAKE’s DEE estimates did not differ significantly from DLW (t(19) = − 1.25; *P* = 0.22), while ACTIREST24’s estimates did (t(19) = − 2.38; *P* = 0.028). Both models underestimated DEE compared to DLW: ACTIWAKE by 14% and ACTIREST by 26%. The inter-individual spread in model estimates of DEE (s.d. 1–2% of mean) was lower than that in DLW (s.d. 33% of mean).

**Conclusions:**

We found that linear locomotion-based calibration applied to the waking period, and a ‘flat’ resting metabolic rate applied to the sleeping period can provide realistic joule estimates of DEE in terrestrial mammals. The underestimation and lower spread in model estimates compared to DLW likely arise because the accelerometer only captures movement-related energy expenditure, whereas DLW is an integrated measure. Our study offers new tools to incorporate body mass (through allometry), and changes in behavioural time budgets and intra-behaviour changes in intensity (through DBA) in acceleration-based field assessments of daily energy expenditure.

**Supplementary Information:**

The online version contains supplementary material available at 10.1186/s40462-023-00395-0.

## Background

Nature imposes a ‘cost of living’ on all animals in the form of energy expenditure, an aspect of daily living as fundamental as position in space or moment in time. Energy expenditure is a key descriptor of fundamental ecological and evolutionary processes such as foraging, dispersal, response to environmental change, health, and fitness. Animals must allocate energy at each instant to various life-history and metabolic requirements, and they meet these requirements by harvesting energy from the environment. Joule estimates of daily energy expenditure (DEE) are required to link an animal’s activity with its energy intake from food using a common currency. There are two laboratory-based methods for measuring energy expenditure: direct calorimetry, where heat generated by the body is directly measured via a thermally sealed chamber, and indirect calorimetry, where heat generation is inferred from the amount of oxygen consumed during respiration [1]. Indirect calorimetry is less expensive and cumbersome than direct calorimetry, but neither can be used in free-living conditions. Consequently, new procedures to measure energy expended by wild-living animals were developed and validated using direct or indirect calorimetry. These procedures include the doubly labelled water (DLW) technique, the ‘heartrate method’, and ‘dynamic body acceleration’ (DBA).

The DLW technique is the gold standard for measuring DEE in free-living animals [2], and provides indirect quantification of respiratory gas exchange. In the DLW technique, a dose of water labelled with heavy stable isotopes ^2^H and ^18^O is injected into the animal; a blood sample is drawn 1–21 days later to estimate the rates of elimination of these isotopes, from which respiratory gas exchange and thereby energy expenditure is inferred [2]. The main asset of the DLW technique is its ability to accurately measure the integrated energetic costs of free-living at the population level [3]. The DLW technique provides energy expenditure estimates that are lumped over several days, have large variation (− 38% to + 54%) in the extent to which they agree with simultaneous indirect calorimetric measurements of individual animals (Table 8.3 in [3]), and may tend to overestimate DEE when compared to indirect calorimetry [4]. In the ‘heartrate method’, heartrate and rate of oxygen consumption are simultaneously measured in the laboratory using a heartrate logger and indirect calorimetry, respectively, to derive a predictive calibration relationship in a first step; subsequently, this calibration equation is applied to heartrate data collected from free-ranging animals to estimate energy expenditure [5]. Validation studies have shown that DEE estimates derived using this method are generally accurate, e.g. within − 28% to + 23% of indirect calorimetric measurements in California sea lions [4], and − 17% to + 19% of DLW measurements in free-living human children [6]. The heartrate method is more invasive than the DLW technique since it requires surgery to implant the heartrate logger [7]; the method poses logistical difficulties because of the inconvenience and cost of the laboratory-based calibration step [8].

DBA is a proxy of energy expenditure computed from acceleration data collected using on-animal accelerometers [9, 10]. Converting these proxies from units of acceleration (m/s^2^) to units of power (W) involves an initial step where a predictive calibration relationship between DBA and rate of oxygen consumption must be derived from laboratory-based respirometry measurements [11, 12]. This technique is noninvasive since it involves accelerometers affixed to the exterior of the body, and allows energy expenditure to be quantified continuously at fine temporal resolution, such as at the scale of seconds. Validation studies with the DLW technique as reference have shown that DEE estimates derived using DBA can have reasonable accuracy, e.g. within − 9% to + 20% in pumas [13], and − 41% to − 16% in polar bears [14]. However, the inherent limitation in this technique is the difficulty of quantifying resting energy expenditure, since DBA is sensitive only to movement-related energy expenditure [10]. This is problematic for DEE estimation, since uncalibrated DBA proxies in m/s^2^ will miss the contribution of energy expended during inactive periods, which can be as long as 20 h in a 24-h period [15]; resting energy expenditure is one of the largest contributors to DEE (see below). Further, similar to the heartrate method, laboratory-based calibration to convert DBA from m/s^2^ to watts is typically costly and impractical for most wild-living species, and there is a lack of general methods to obtain realistic substitutes for empirical calibration relationships when these are unavailable.

A wealth of past allometric work offers a ‘database’ for deriving suitable estimates of rate of energy expenditure in standard units (W). Two well-known examples are Kleiber’s law for estimating resting metabolic rate from body mass [16], and work by Taylor and colleagues to estimate the cost of terrestrial locomotion from body mass and speed [17]. This offers the opportunity to derive practical species-specific calibration relationships by linking allometric estimates to DBA, but how exactly to do so is not obvious. Empirical calibration relationships for a given species are often linear (e.g. [9, 11, 18]). Single straight-line calibration equations perform well even when the species has multiple gaits, such as walking and running in humans, even though accuracy improves when the model is made more complex by performing regression separately for each gait [19]. Deviations from single-line calibration equations have mainly been considered in animals with habitats spanning multiple media, e.g. birds traversing air and water (e.g. [20, 21]), or mammals traversing land and water (e.g. [14].). Here, we consider the relatively simpler case of land-based animals for which a linear calibration equation seems a good compromise between accuracy and complexity.

A simplistic expectation from a linear calibration equation would be that the vertical intercept (rate of energy expenditure at zero DBA) should approximate resting metabolic rate. However, empirical studies have shown that this is not true in practice. For instance, intercepts can differ from resting metabolic rate by more than a factor of two [22], or indeed be negative [23]. Further, a comparison of DBA with the heart rate method showed that while the accuracy of the two methods was similar for active behaviours, errors in acceleration-based estimates were larger for inactive behaviours [24]. The difficulty in accurately capturing energy expenditure during both resting and activity using a single linear equation suggests an inherent nonlinearity. One cause of this nonlinearity might be the increase in internal body temperature arising from activity. For instance, in a study on the energetics of running in mammals [25], the authors found that rectal temperature increased with increasing running speed. They argued that this might explain why their vertical intercept (time rate of energy expenditure at zero speed) was greater than resting metabolic rate. This is especially relevant for DEE estimation, since resting metabolic rate is one of the two largest contributors (30–60%) to DEE, the other being the energetic cost of physical activity (25–87%) [26–29]. It is thus worth investigating how a calibration model should treat activity and inactivity: separately, or together in a single equation.

In this study, we address these questions by developing two classes of linear calibration equations that link DBA with allometric energy expenditure. One model (ACTIWAKE) treats the waking and sleeping periods separately: the calibration equation is developed from locomotion data and applied during the waking hours when the animal is active, while resting metabolic rate is estimated separately and applied during the sleeping period. In the other model (ACTIREST24), the calibration equation is developed from both locomotion and resting data and applied to the entire 24-h period. Both calibration equations convert DBA from m/s^2^ to W, and their application to 24-h acceleration yields estimates of daily energy expenditure in joules. Since both models require information on locomotion speed, we develop a new method to estimate speed from accelerometer and gyroscope data, and behavioural annotation of videos available from a previously reported dataset. We apply these methods to estimate DEE in wild-living meerkats (*Suricata suricatta*). Finally, we compare DEE estimates obtained from the three models with published DEE measurements made using doubly labelled water in the same population of meerkats.

## Methods

### General framework for calibrating dynamic body acceleration using allometry for daily energy expenditure estimation

Our approach centres on using allometric estimates of behaviour-specific energy expenditure as a viable substitute for laboratory-based respirometry measurements when the latter are unavailable. Similar to respirometry studies where in-lab calibration measurements of specific behaviours, e.g. locomotion, enable the conversion of DBA to energy expenditure via a linear relationship (e.g. [9, 18]), we considered allometric energy expenditure and corresponding values of vectorial DBA to be linearly related through an analogous ‘power calibration equation’. We use two allometric equations to capture the effects of the biggest contributors to daily energy expenditure: resting and physical activity. We use Kleiber’s law [16] to estimate resting metabolic rate ($\dot{E}_{R}$, in W) from body mass ($M$, in kg) (Eq. 1):

1 $$\dot{E}_{R} = 3.477M^{0.75}$$

We consider locomotion as an archetypal non-resting behaviour representative of physical activity, and use Taylor, Heglund, and Maloiy’s 1982 data [17] to estimate the rate of energy expenditure during locomotion ($\dot{E}_{L}$, in W) given body mass and speed ($v$, in m/s). Their multi-species equation (Eq. 2) is given by:

2 $$\dot{E}_{L} = 10.7{\text{M}}^{0.684} .v + 6.03M^{0.697}$$

We selected these two studies because they provide simple equations that capture the body mass-dependence of energy expenditure for two key behaviours, resting and locomotion, and because the large size range and diversity of species on which their allometric equations are based allow for the generalisation of our approach to a variety of species. Kleiber’s study on resting metabolism considered eight mammalian and avian species in the size range 0.15–679 kg [16]. Taylor et al.’s study considered terrestrial locomotion energetics in 62 mammalian and avian species, both bipeds and quadrupeds, in the size range 0.021–254 kg [17]. Broadly speaking, it is therefore reasonable to expect our approach to be valid for the intersection of these two datasets, i.e. land-based mammalian species in the size range 0.15–254 kg. Equations 1 and 2 represent ‘cross-species averages’ that are agnostic to the precise species under consideration, and we draw the reader’s attention to the fact that Table 1 in [16] and Table 1 in [17] contain species-specific equations for computing $\dot{E}_{R}$ and $\dot{E}_{L}$, respectively. We advocate that when implementing the proposed method, researchers use appropriate species-specific equations whenever possible—either for the very same species if available or for a surrogate species—to ensure that power estimates are as accurate as possible. Given that studies [16] and [17] pool data from multiple individuals to build their species-specific equations, the appropriate interpretation of the resulting values of $\dot{E}_{R}$ and $\dot{E}_{L}$ would be that these are group-level averages for the species of the given body mass; the equations are unlikely to accurately represent intraspecific differences in energy expenditure arising from differences in body mass alone.

In the proposed power calibration procedure, we link vectorial DBA $a_{d}$ during resting and locomotion—calculated from triaxial acceleration in m/s^2^ according to [18] over windows of fixed length (say two seconds)—to power values $\dot{E}_{R}$ and $\dot{E}_{L}$, respectively. Implicit here is the requirement of the following information:

1. *Acceleration collected during resting and locomotion behaviours*
  Studies aiming at inferring behaviour from acceleration data already collect time-synchronised behavioural data of free-ranging tagged animals either through visual observation [30] or video-based annotation [31] to compile a groundtruthed dataset that is used to train machine learning algorithms that classify snippets of acceleration data into behaviours of interest. Labelled acceleration corresponding to resting and locomotion behaviours can be obtained from such training datasets.
2. *Body mass*
  Body mass of individual animals can be, and often is, measured in the field during the tagging process.
3. *Locomotion speed*
  Locomotion speed can be estimated using additional sensors commonly present in biologging tags, for instance, either a GPS sampling at high frequency (say at ≥ 1 Hz) or an inertial measurement unit (IMU) coupled with algorithms such as the Kalman filter to fuse accelerometer and gyroscope data (e.g. [32]; see our speed estimation algorithm below).

**Fig. 1:**
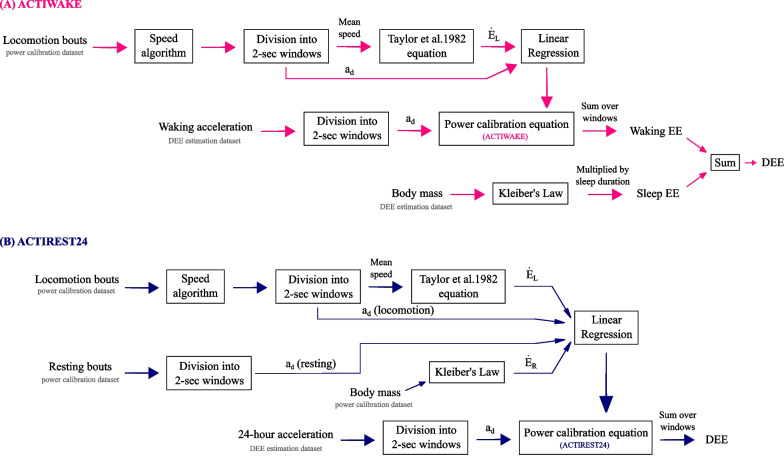
**Two models for estimating daily energy expenditure in meerkats.** We developed two models to estimate DEE in meerkats. Both involve computing a linear ‘power calibration equation’ that converts vectorial dynamic body acceleration (${a}_{d}$, in m/s^2^) to power in watts using allometric energy expenditure. We computed rate of energy expenditure during resting (${\dot{E}}_{R}$, in W) using Eq. 1 [16], and that during locomotion (${\dot{E}}_{L}$, in W) using Eq. 3 [17]. In ACTIWAKE (**A**), the waking and sleeping hours were treated separately. The power calibration equation was computed using locomotion data and applied during the waking period; the animal was assumed to be resting during the sleeping period. Daily energy expenditure (DEE) was computed as the sum of waking and sleeping energy expenditure. In ACTIREST24 (**B**), no distinction was drawn between the waking and sleeping hours. The power calibration equation was computed using both locomotion and resting data and applied to the entire 24-h period. Both **A **and **B** required information on locomotion speed. For this, we developed a new algorithm (Fig. 2) to infer speed from accelerometer, gyroscope, and behavioural annotation data. EE: energy expenditure

We considered two models to estimate DEE:

(i) ACTIWAKE (Fig. 1A): here, we use information from locomotion bouts to derive the power calibration equation, and treat the waking and sleeping periods differently:
  - *Waking period*: we calculate ${a}_{d}$ in m/s^2^ according to [18] over sliding windows (see Sect. 2.2 for details), and convert these values to $\dot{E}$ using the power calibration equation. We multiply $\dot{E}$ by window length, and sum these products over windows to obtain the energy expended while awake in joules.
  - *Sleeping period*: we calculate ${\dot{E}}_{R}$ using Eq. 1 and multiply it by the duration of the sleeping period to obtain the energy expended while asleep in joules.
  We sum energy expended during the waking and sleeping periods to obtain DEE. Though these principles would apply regardless of the precise sleep schedule (i.e. diurnal, nocturnal, crepuscular, cathemeral, or other), this method requires timing and duration of sleep to be known. Information on sleep schedules can be obtained either through manual observation of animals when possible, or by using algorithms to infer sleep schedule from on-animal accelerometer data (e.g. [33, 34]).
(ii) ACTIREST24 (Fig. 1B): here, we use information from both locomotion and resting bouts to derive the power calibration equation. We then apply this equation over the entire 24-h period—similar to the operations described for computing energy expended during the waking period in the ACTIWAKE model—to compute DEE in joules.

### Application to Kalahari meerkat data

#### Data collection

We conducted fieldwork at the Kalahari Meerkat Project [35]. The dataset used in this study consisted of two parts:

(i) *Power calibration dataset*
  We used the dataset described in [36] for deriving power calibration relationships from IMU data (details in later subsections). Briefly, we collected data on ten adult meerkats (seven males, three females) from August 2016 to November 2017 using collars of size 35 mm × 29 mm × 19 mm, and mass < 25 g (Physilog IV, GaitUp SA, Switzerland). Collars housed a triaxial accelerometer (range ± 16 g; g = 9.81 m/s^2^) and triaxial gyroscope (range ± 2000 deg/s), both recording at 100 Hz/axis with 16-bit resolution. Collared animals were filmed for around 3 h in the morning using a handheld video camera recording at 25 frames/second and synchronised electronically with the IMU as described in [37]. Videos were annotated using Solomon Coder (Version: beta 17.03.22).
(ii) *DEE estimation dataset*
  We used the dataset described in [38] (Chapter 6 therein) for estimating DEE in meerkats using acceleration data (details in later subsection). Briefly, we collected 24-h accelerometer data (Table 1) on ten adult meerkats (five females, five males) from April 2018 to August 2019 using collars of size 37 mm × 22 mm × 22 mm, and mass < 25 g (CDD Ltd, Athens, Greece). Collars housed a triaxial accelerometer recording at 50 Hz/axis with a range of ± 8 g at 16-bit resolution throughout the day for 1–6 days per individual (Table 1).

Individuals were captured following methodology described in [39]. While an animal was anaesthetised, its body length (base of neck to base of tail) was measured using vernier callipers. Collars of the mentioned size and mass do not affect meerkat behaviour [40]. We measured animal body mass by enticing individuals to stand on an electronic platform balance, for which they had been previously trained [41]. For consistency, we used morning body mass, i.e. measured before the start of the day’s foraging, in energetics calculations.

**Table 1:** Summary of DEE estimation dataset

Individual #	Sex	Age (months)	Body mass (g)	# days of data	Month and year
1	F	25	581 ± 14	6	Aug–Sep 2018
2	F	25	537 ± 6	5	Aug–Sep 2018
3	F	22	627 ± 9	6	Sep 2018
4	F	12	577 ± 10	6	Sep 2018
5	F	12	513 ± 2	4	Jul 2019
6	M	30	NA	6	Jul 2019
7	M	48	733 ± 10	3	Jul 2019
8	M	16	660 ± 4	5	Aug 2019
9	M	16	600 ± 1	2	Aug 2019
10	M	23	717	1	Aug 2019

Our DEE estimation dataset comprised of a total of 44 days of accelerometer data collected over 2 years (2018–2019) during the winter months of Jul–Sep from ten adult individuals, all subordinates in their respective groups (five females, five males). Body mass was available for 31 of these days for all individuals except #6

We used findings from long-term studies of the same population of Kalahari meerkats to inform our estimation of meerkat sleep schedules. Meerkats are diurnal group-living animals that retreat to underground burrows to sleep shortly after sunset, with different groups emerging from their sleeping burrows around sunrise within ± 15 min of each other [42]. We used sunrise and sunset times as proxies for awakening and sleep-onset times, respectively, and assumed that individuals were not active during the night inside their sleeping burrows. The latter assumption is supported by ongoing work by Chakravarty et al., where night-time accelerometer data indicates a sleep efficiency (total time spent inactive divided by time elapsed between accelerometer-inferred sleep onset and awakening) of > 95%. We used the suncalc R package (suncalc::getSunlightTimes) [43] to compute precise sunrise and sunset times at the Kalahari Meerkat Project (26.96 S, 21.83 E), following [44]. Ethical approval to conduct this research was granted by the Mammal Research Institute, University of Pretoria (permit no. FAUNA 1020/2016).

We calibrated the accelerometer prior to deployment on the animal using the ‘tumble test,’ which uses gravitational acceleration as reference, following [45]. Here, we affixed the accelerometer to the inner wall of a rigid cuboidal box such that two of the axes of the accelerometer were parallel to the sides of the inner wall. We placed each of the six outer walls of the box face-down and motionless on a flat level surface for a few seconds one after the other, and repeated the whole sequence twice. This procedure aligns each axis of the triaxial accelerometer along the vertical direction four times: twice along + 1 g, twice along − 1 g (0 g in other orientations). We computed the offset and gain of each axis, and calibrated meerkat acceleration data by subtracting the corresponding axis’ offset and dividing the result by the axis’ gain.

#### Algorithm for speed estimation using IMU data

**Fig. 2:**
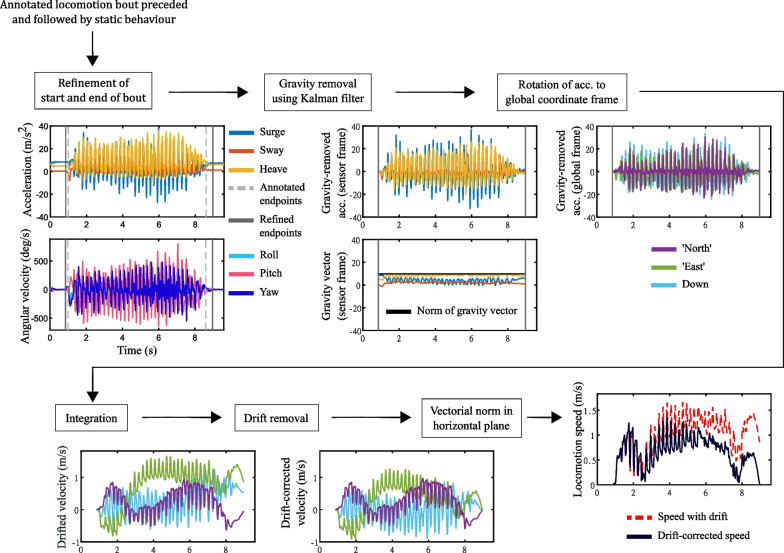
**Speed estimation from accelerometer, gyroscope, and behavioural annotation data.** We developed an algorithm to estimate locomotion speed from IMU data and behavioural annotation. We used the behavioural annotation in our power calibration dataset to select locomotion bouts preceded and followed by static behaviour (resting or vigilance). We refined the start and end of these locomotion bouts by using acceleration and angular velocity thresholds to ensure that starting and ending velocity and acceleration were zero. We removed gravitational acceleration by fusing accelerometer and gyroscope data in an internal error-state Kalman filter, and rotated gravity-compensated acceleration from the sensor frame to the global coordinate frame using quaternions obtained by applying the function imufilter in MATLAB. We integrated acceleration in the global coordinate frame to obtain velocity and removed integration drift through linear compensation using the information that initial and final velocity are zero. Finally, we computed the vectorial norm of the velocity components in the horizontal plane to obtain the instantaneous locomotion speed signal. Shown here is a bout of running preceded and followed by vigilance

Using the power calibration dataset, we combined video-based behavioural annotation with triaxial accelerometer and triaxial gyroscope data to estimate locomotion speed (Fig. 2). Our algorithm is an adaptation of well-known methods developed and validated for human gait analysis (e.g. [32]), and we present it in detail here to help facilitate uptake of these state-of-the-art methods from human gait research by the movement ecology research community. Integration of acceleration to obtain velocity from IMU data usually requires (1) initial acceleration to be zero, and (2) drift compensation, which requires final velocity to be known. To meet these requirements, we used our behavioural annotation to select bouts of locomotion (walking or running) that were preceded and followed by static behaviour (resting or vigilance). This had the advantage of ensuring that both initial acceleration and final velocity are zero, because the animal is stationary during static behaviour. However, since it is difficult to visually pinpoint the precise moment the animal starts and finishes a locomotion bout, we developed an algorithm to refine the annotated starting and ending times (*a*_*1*_ and *a*_*2*_, respectively) of locomotion bouts.

First, we filtered data from each axis of the accelerometer and gyroscope with a low-pass Butterworth filter of order 4 and cut-off frequency 25 Hz (using the function butter in MATLAB R2022a; all analyses were performed in MATLAB). We then used thresholds based on the standard deviation of the vectorial norm of acceleration and angular velocity (0.02 g and 5 deg/s, respectively) computed in a short running window of duration 0.1 s to select ‘stationary regions’ of the signal in a one-second ‘refinement interval’ around *a*_*1*_ and *a*_*2*_. The last instant within the refinement interval around *a*_*1*_ that still belonged to a stationary region was selected to be the refined starting moment of the locomotion bout *t*_*1*_. Similarly, the first instant within the refinement interval around *a*_*2*_ that still belonged to a stationary region was selected to be the refined ending moment of the locomotion bout *t*_*2*_. We removed any locomotion bouts where *t*_*1*_ and *t*_*2*_ could not be found because the threshold criteria could not be met.

Next, we integrated acceleration in the global coordinate frame to obtain velocity. Our algorithm involved five steps:

1. We fused accelerometer and gyroscope data in the interval *t*_*1*_ to *t*_*2*_ in an internal error-state Kalman filter using the function imufilter (default parameters, with ‘North-East-Down’ reference frame) based on [46] to compute quaternions describing the instantaneous orientation of the sensor frame relative to the global coordinate frame. Note that imufilter assumes that the device’s first axis is initially pointing northwards [47], which may not correspond to the actual heading of the animal. However, since we were interested in locomotion speed rather than direction, absolute heading was irrelevant.
2. We used the orientation quaternions to compute the instantaneous gravitational acceleration vector in the sensor frame, and subtracted the latter from total acceleration to obtain gravity-compensated acceleration in the sensor frame.
3. We rotated gravity-compensated acceleration from the sensor frame to the global coordinate frame using the orientation quaternions (Fig. 2, top-right panels).
4. We integrated acceleration in the global coordinate frame. For this, we set initial velocity at *t*_*1*_ to zero. Since final velocity at *t*_*2*_ is also zero, any difference from zero must arise due to integration drift. We distributed the drift linearly in the interval *t*_*1*_ to *t*_*2*_ such that final velocity was zero.
5. We computed the vectorial norm of the velocity components in the horizontal plane in the global coordinate frame to obtain instantaneous locomotion speed for the bout (Fig. 2, bottom panels).

We have provided code to perform gravity compensation (gravRemov_imufilter.m), and compute domain of integration of acceleration and locomotion speed (speed_strapdownIntegration.m) in a GitHub repository accompanying this work [48].

#### Power-calibrated estimation of daily energy expenditure in meerkats

To obtain the power calibration equations, we calculated four quantities—${a}_{d}$, $v$, $M$, and $\dot{E}$—from the power calibration dataset. We removed the first and last 0.5 s of each locomotion bout (relative to the bout’s *t*_1_ and *t*_2_) as we considered these to be ‘transition periods’ where the animal was speeding up from and slowing down to a stationary position, respectively. We divided the remainder of each locomotion bout into two-second windows with 50% overlap between successive windows. We computed vectorial dynamic body acceleration (${a}_{d}$, in m/s^2^) and mean locomotion speed $v$ (in m/s) for each window. Since data from dwarf mongooses was available in [17], and because both species are mongooses that have similar body mass and locomotion styles and can function as surrogate species for each other, we used the dwarf-mongoose equation (Eq. 3) to compute $\dot{E}_{L}$ for meerkats.

3 $$\dot{E}_{L} = 13.467{\text{M}}v + 7.236M$$

We computed $\dot{E}_{R}$ using Eq. 1. We performed linear regression using fittype and fit (MATLAB® R2022a). We developed two DEE-prediction models (Fig. 1):

(i) ACTIWAKE: here, we computed $\dot{E}_{L}$ and $a_{d}$ values from locomotion bouts, and performed linear regression to relate $\dot{E}$ to $a_{d}$.
(ii) ACTIREST24: here, we computed $\dot{E}_{L}$, $\dot{E}_{R}$ and $a_{d}$ values from locomotion and resting bouts, and performed linear regression to relate $\dot{E}$ to $a_{d}$. With $n_{w}$ denoting the number of two-second windows of locomotion in the power calibration dataset, we used random sampling to choose an equal number $n_{w}$ of two-second windows of resting and computed resting $a_{d}$ from these.

We have provided code to implement the ACTIWAKE and ACTIREST24 models for meerkats (dee_actiwake_actirest24.m) in a GitHub repository accompanying this work [48].

#### Comparison of model estimates of DEE with doubly labelled water estimates of DEE, and statistical analysis

We compared DEE predictions with published DEE measurements made using the doubly labelled water (DLW) technique in the same population of meerkats [49, 50] (Additional file 1: Appendix S1) using the two-sample t-test (ttest2 in MATLAB® R2022a) (Table 2). All individuals compared in this and the DLW studies were subordinate and non-lactating and were measured in the same season (winter).

**Table 2:** Comparison of model estimates of DEE with doubly labelled water measurements in meerkats

DEE estimation method	Females		Males		Females + males	
	DEE (kJ)	Mass (g)	DEE (kJ)	Mass (g)	DEE (kJ)	Mass (g)
DLW	468 ± 89 (321–521) N = 6	697 ± 68 (632–799)	576 ± 223 (278–941) N = 6	749 ± 131 (591–922)	522 ± 172 (278–941) N = 12	723 ± 103 (591–922)
ACTIWAKE	440 ± 4 (437–445) N = 5 (23 days acc) t(9) = − 0.68; *P* = 0.51	560 ± 42 (507–615) t(9) = − 3.90; *P* = 0.004	461 ± 11 (448–471) N = 4 (8 days acc) t(8) = − 1.01; *P* = 0.34	670 ± 61 (593–721) t(8) = − 1.10; *P* = 0.30	449 ± 13 (437–471) N = 9 (31 days acc) t(19) = − 1.25; *P* = 0.22	609 ± 75 (507–721) t(19) = − 2.79; *P* = 0.012
ACTIREST24	389 ± 7 (381–399) N = 5 (23 days acc) t(9) = − 1.95; *P* = 0.082	as above	379 ± 9 (366–385) N = 4 (8 days acc) t(8) = − 1.73; *P* = 0.12	as above	384 ± 9 (366–399) t(19) = − 2.38; *P* = 0.028	As above

We found that ACTIWAKE’s estimates of daily energy expenditure (DEE) did not differ significantly from measurements made using the doubly labelled water method (DLW), while ACTIREST24’s estimates did (results of two-sample t-test in table). Both models underestimated DEE compared to DLW: ACTIWAKE by 14% (females and males pooled together), and ACTIREST24 by 26%. DLW measurements had greater spread (s.d. 33% of mean; all individuals pooled together) compared to ACTIWAKE’s estimates (3%). Published DLW data were available for 12 individuals (six females, six males). Our DEE model estimates were derived from 31 days of acceleration data collected from nine individuals (five females, four males)

## Results

### Locomotion speed estimation

In the power calibration dataset (Sect. 2.2, Data collection), we found nine walking bouts (4.1 ± 1.9 s) and nine running bouts (5.7 ± 2.0 s) that were preceded and followed by static behaviour. Our speed estimation algorithm yielded a mean bout speed of 0.35 ± 0.16 m/s for walking and 0.85 ± 0.17 m/s for running. Using a separate method, we found that these speed values were realistic (details of speed assessment method and results are in Additional file 1: Appendix S2).

### Power calibration

Morning body mass was available for 12 of the 18 locomotion bouts in the power calibration dataset. These yielded 20 two-second windows of locomotion: seven of walking and 13 of running. We obtained the following equation (Eq. 4) for the ACTIWAKE model:

4 $$\dot{E}\,\left[ {\text{W}} \right] = 0.641\left[ {\frac{{{\text{Js}}}}{{\text{m}}}} \right]a_{d} \left[ {\frac{{\text{m}}}{{{\text{s}}^{2} }}} \right] + 6.443\left[ {\text{W}} \right]\quad \left( {R^{2} = 0.63} \right)$$

With the addition of an equal number (i.e. 20) of randomly chosen two-second windows of resting behaviour, we obtained the following equation for the ACTIREST24 model:

5 $$\dot{E}\left[ {\text{W}} \right] = 0.906\left[ {\frac{{{\text{Js}}}}{{\text{m}}}} \right]a_{d} \left[ {\frac{{\text{m}}}{{{\text{s}}^{2} }}} \right] + 3.104\left[ {\text{W}} \right]\quad \left( {R^{2} = 0.82} \right)$$

### DEE estimation

The DEE estimation dataset (Sect. 2.2, Data collection) consisted of 44 days of acceleration data (Table 1). We estimated DEE from 31 of these days where morning body mass was available (all individuals except #6). ACTIWAKE’s DEE estimates did not differ significantly from DLW when all individuals were pooled together, while ACTIREST24’s estimates did (Table 2; t-test results within table). Compared to DLW, ACTIWAKE’s DEE estimates were 6% smaller for females, 20% smaller for males, and 14% smaller when females and males were pooled together (Table 2). In comparison, these percentages were 17%, 34% and 26% for ACTIREST24, respectively. While ACTIWAKE produced DEE estimates that were smaller for females than for males (t(7) = − 4.07; *P* = 0.005) as would be expected from the lower body mass of females compared to males in this study (t(7) = − 3.22; *P* = 0.015), ACTIREST24 displayed the opposite trend by producing larger DEE estimates for females compared to the males, although this was not statistically significant (t(7) = 1.81; *P* = 0.11). We remarked that individuals in the current study were in general lighter than those in the DLW studies: females by 20% and males by 11% (Table 2; t-test results within table). DLW measurements had greater spread compared to ACTIWAKE’s estimates: standard deviation was 19% of the mean for females and 39% for males for DLW, while these percentages were 1% and 2% for ACTIWAKE, respectively.

## Discussion

We presented a framework to estimate daily energy expenditure in terrestrial mammals that is based on calibrating DBA using allometric estimates of energy expenditure. This offers a practical solution to the issue of converting acceleration proxies of energy expenditure from units of m/s^2^ to standard units (watts) when in-lab respirometry or calorimetric measurements are unavailable. We developed two ‘power calibration’ models and used these to estimate DEE in wild-living meerkats, and compared our models’ estimates with measurements based on doubly labelled water. Since these models require speed information, we developed a new algorithm for high-resolution inference of speed from accelerometer, gyroscope, and behavioural annotation data. We found that model estimates of DEE based on a linear power calibration equation were in agreement with doubly labelled water measurements of DEE when the waking and sleeping hours were treated differently (ACTIWAKE), but not when a single equation was used across the 24-h period (ACTIREST24).

In this study, our focus was on developing a practical alternative to lab-based respirometry calibrations that provides realistic DEE estimates by harnessing allometric literature on terrestrial mammals. Our goal was not to capture all factors contributing to DEE; indeed, DBA has inherent limits, such as the inability to account for non-movement-related factors such as thermogenesis [10], that make comprehensiveness unlikely for any acceleration-based model. Nevertheless, a linear calibration-based model (ACTIWAKE) provided realistic results in this study, and more generally has the advantage of having higher ‘energetic resolution’ and being more parsimonious than other approaches such as time-energy budget (TEB) models [20]. TEB models are based on estimating DEE by summing the energetic cost of each behaviour (e.g. in the form of constant multiples of resting metabolic rate) weighted by the durations of these behaviours. Constant energetic cost for a given behaviour cannot account for intra-behaviour variations in energy expenditure, while a value of energetic cost must be assigned to each behaviour in the ethogram, which increases the number of variables in the model. On the other hand, a calibration-based model has near-instantaneous energetic resolution since it converts changes in DBA occurring every few seconds (two seconds in this study) to changes in energy expenditure independently of behavioural category, and typically uses information from only locomotion and resting to derive energy-cost estimates for other behaviours. The same concept, but using an empirical laboratory-to-field approach, has been demonstrated successfully in wild pumas (*Puma concolor*): respirometry measurements during treadmill running and resting were used to derive a linear calibration relationship, which was then used to estimate energy expenditure during non-locomotion dynamic behaviours such as feeding and grooming [22, 51].

**Fig. 3:**
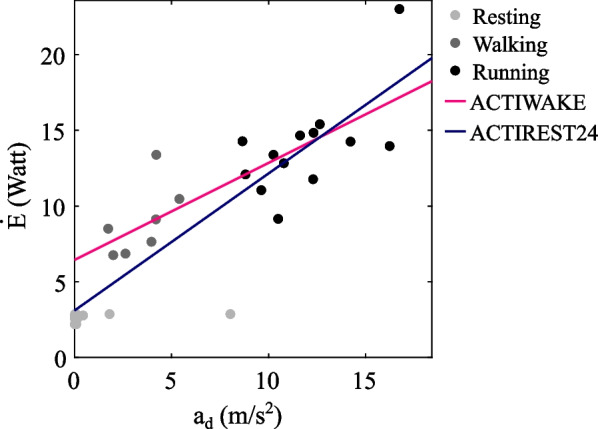
**Linear power calibration.** Using our power calibration dataset, we considered two linear models to calibrate vectorial dynamic body acceleration (${a}_{d}$, in m/s^2^) using allometric estimates of energy expenditure ($\dot{E}$, in W) during resting (Eq. 1) and locomotion (Eq. 3). In the ACTIWAKE model (in pink; smaller slope and larger intercept), we performed linear regression using data from locomotion (walking, running) while for the ACTIREST24 model (in blue; larger slope and smaller intercept), we used data from both locomotion and resting

Comparing our two models, ACTIWAKE’s intercept was more than double ACTIREST24’s intercept, while ACTIREST24’s slope was 1.4 times ACTIWAKE’s slope (Eqs. 4 and 5). Consequently, ACTIWAKE yielded larger $\dot{E}$ at lower $a_{d}$ (< 12.6 m/s^2^) while ACTIREST24 yielded larger $\dot{E}$ at higher $a_{d}$ (≥ 12.6 m/s^2^) (Fig. 3). The closer agreement of ACTIWAKE with DLW compared to that of ACTIREST24 would imply that higher baseline $\dot{E}$ during the waking hours was more important for ensuring realistic DEE estimates for meerkats than higher rates of increase of $\dot{E}$ with $a_{d}$. One reason why both models underestimated DEE compared to DLW could be the lower body mass of individuals in this study compared to the DLW study. An additional reason could have been that DLW-derived DEE is an integrated measure that takes into account effects as varied as thermoregulation and differences in body tissue composition [3], whereas DBA primarily quantifies sensor motion. This might also explain why variation in DLW measurements across individuals may have been higher than in the acceleration models. We speculate that the temperature effects of physical activity [25] could also have contributed to the underestimation of energy expenditure. For instance, while DBA will instantly drop when a bout of vigorous activity (e.g. running) ends and is followed by static behaviour (e.g. resting), energy continues to be expended for some time at higher rates than during resting, a phenomenon termed ‘excess postexercise energy expenditure’ in human studies [52].

ACTIREST24’s DEE estimates were 26% smaller than DLW, which is very similar to the 30% underestimation of DEE using an ACTIREST24-like respirometry-based linear calibration model combining data collected during treadmill resting and locomotion in free-ranging polar bears (*Ursus maritimus*) [14]. We observed that the intercept of their linear calibration model for nonswimming behaviours (their Fig. 2) was < 0.1 ml O_2_ g^−1^ h^−1^ whereas the resting metabolic rate used to construct the model was 0.230 ml O_2_ g^−1^ h^−1^—this implies that their model would underestimate the energetic cost of resting by more than a factor of two. One caveat in the comparison between the two studies is the ± 2 g range of their accelerometers, which we suspect gets saturated during dynamic behaviours, thereby leading to smaller values of DBA than would otherwise be measured by accelerometers having a larger range (± 8 g or more, as in this study). Nevertheless, given that polar bears spend most of their time (> 85%) resting even during summer [53], decoupling the waking and sleeping periods in the application of the power calibration equation would likely have increased the accuracy of their acceleration-based DEE prediction. For the meerkats in our study, decoupling the waking and sleeping periods in the ACTIWAKE model led to a significantly smaller DEE underestimation of 14% compared to DLW.

The allometric equations used here are based on aggregate interspecific measurements, and are usually understood to provide an average value of energy expenditure for a specific species. This is why, for instance, the equation specific to dwarf mongooses (Eq. 3) sourced from [17] does not consider energy expenditure to vary with intraspecific variations in body mass. However, combining allometric estimates with DBA, which can vary from one window to the next, allows fine-scale ‘modulation’ of the average species-specific value by the particular behavioural dynamics of the animal. Since different behaviours typically correspond to a characteristic range of DBA values (see Additional file 1: Appendix S3 for behaviour-specific DBA for meerkats), this approach would be able to quantify, in joules, fine-scale energetic differences associated with differences in behavioural time budgets. Some examples of important downstream consequences of this include the ability to quantify the effects of external factors such as food availability [54], habitat [55], and group size [56], since behavioural time budgets have been shown to change in response to changes in these factors. Thus, this ‘hybrid’ approach involving the modulation of coarse-scale allometry by fine-scale DBA allows for detailed intraspecific and intraindividual comparisons. The level of detail of such approaches could be further enhanced through the use of intraspecific (e.g. for humans [57, 58]) and intraindividual (e.g. for kestrels [59]) allometric equations.

## Conclusions

We presented a simple procedure to leverage existing allometric work to calibrate dynamic body acceleration when empirical respirometry-based calibration is unavailable. The use of allometry in our method allows the incorporation of body-mass effects, while the use of fine-scale DBA allows sensitivity to changes in behavioural time budgets and intra-behaviour changes in intensity. Our results indicate that linear calibration equations can provide realistic estimates of DEE, provided the waking and sleeping periods are treated separately. By offering a practical ‘literature-to-field’ alternative to ‘laboratory-to-field’ calibration, our study stands to supplement the utility and generality of acceleration-based field studies of animal energetics.

### Supplementary Information

**Additional file 1.** This file contains three appendices. **Appendix S1** presents published measurements of daily energy expenditure in meerkats made using the doubly labelled water technique. **Appendix S2** presents an assessment of whether speed estimated using our IMU-based algorithm was realistic. **Appendix S3** presents vectorial dynamic body acceleration for different meerkat behaviours computed using the power calibration dataset.
