# Correction: Combining accelerometry with allometry for estimating daily energy expenditure in joules when in-lab calibration is unavailable

**DOI:** 10.1186/s40462-023-00422-0

**Published:** 2023-09-27

**Authors:** Pritish Chakravarty, Gabriele Cozzi, David Michael Scantlebury, Arpat Ozgul, Kamiar Aminian

**Affiliations:** 1 Institute of Bioengineering, Ecole Polytechnique Fédérale de Lausanne, Lausanne, Switzerland; 2 Department of Evolutionary Biology and Environmental Studies, Universität Zürich, Zurich, Switzerland; 3 Department for the Ecology of Animal Societies, Max Planck Institute of Animal Behavior, Constance, Germany; 4 Kalahari Research Centre, Kuruman River Reserve, Van Zylsrus, 8467 South Africa; 5 School of Biological Sciences, Queen’s University Belfast, Belfast, Northern Ireland, UK

**Correction:** ***Movement Ecology*** **(2023) 11:29**

10.1186/s40462-023-00395-0

Following publication of the original article [1], the authors identified an error in one of the cells in Table 2 due to a typesetting mistake: in the last row middle column under “DEE (kJ)” for “Males”, the entry was incorrectly captured as “9 ± 9”, whereas the correct entry should be “379 ± 9”.

The original article [1] has been corrected and the publisher apologises to the authors and readers for the inconvenience caused by these errors.
