# Supplementary material for: Combining accelerometry with allometry for estimating daily energy expenditure in joules when in-lab calibration is unavailable
Source: Mov Ecol. 2023 May 30;11:29. doi: 10.1186/s40462-023-00395-0 (PMC10228015; doi:10.1186/s40462-023-00395-0)
Supplement: Supplementary file 1 — Additional file 1. This file contains three appendices. Appendix S1 presents published measurements of daily energy expenditure in meerkats made using the doubly labelled water technique. Appendix S2 presents an assessment of whether speed estimated using our IMU-based algorithm was realistic. Appendix S3 presents vectorial dynamic body acceleration for different meerkat behaviours computed using the power calibration dataset. [file 40462_2023_395_MOESM1_ESM.docx]

# Appendix S1 | Published measurements of daily energy expenditure in meerkats made using the doubly labelled water technique

| # | DEE (kJ/day) | Body mass (grams) |
| --- | --- | --- |
|  |  |  |
| *Subordinate females* | | |
| 1 | 321 | 667 |
| 2 | 528 | 632 |
| 3 | 541 | 693 |
| 4 | 494 | 633 |
| 5 | 396 | 755 |
| 6 | 526 | 799 |
|  |  |  |
| *Subordinate males* | | |
| 7 | 941 | 675 |
| 8 | 467 | 763 |
| 9 | 497 | 660 |
| 10 | 669 | 591 |
| 11 | 278 | 880 |
| 12 | 604 | 922 |

Table S1. **Individual-wise** **daily energy expenditure values of meerkats determined using the doubly labelled water technique (Scantlebury et al. 2002, 2004).** Measured daily energy expenditure values were used as a reference for comparison with predicted DEE.

# Appendix S2 | Assessment of whether speed estimated using our IMU-based algorithm was realistic

To assess whether our IMU-based speed estimates were realistic, we compared mean estimated speed during running bouts computed using our algorithm with the product of body length and dominant frequency of the corresponding acceleration signal (‘FFT-based speed’). Visual inspection of videos indicated that the animal would cover roughly one body length per bound during running (we excluded walking in this part because distance covered per stride was shorter, which made it difficult to find a general distance reference). Further, we reasoned that dominant frequency of the acceleration signal would reflect the average number of bounds per second over the running bout. For each running bout in the range *t_1_* to *t_2_*, we computed mean estimated speed, and computed dominant frequency using getFFTpeakpower, code developed previously by Chakravarty et al. 2019.

We found that the absolute relative difference between IMU- and FFT-based speed for running bouts was 12.9 ± 7.3 %. There was one outlier ($-$29.5%) from a bout where videos showed bounds in the latter half to be very short, unlike other observed bounds in the same and other bouts. Since bound length was assumed to be one body length, the FFT-based method overestimated running speed in this instance. Without this outlier, absolute relative difference between the two methods reduced to 10.6 ± 3.2 %. Similarly, the largest remaining difference (+15%) occurred in a bout where videos showed the meerkat to be running faster (mean IMU-based speed 1.17 m/s, 14% faster than the next fastest bout) with noticeably longer bounds than in any of the other bouts: the FFT-based method likely underestimated running speed in this instance. Without this bout, absolute relative difference further reduced to 9.8 ± 2.8 %.

# Appendix S3 | Vectorial dynamic body acceleration for different meerkat behaviours, computed using the power calibration dataset


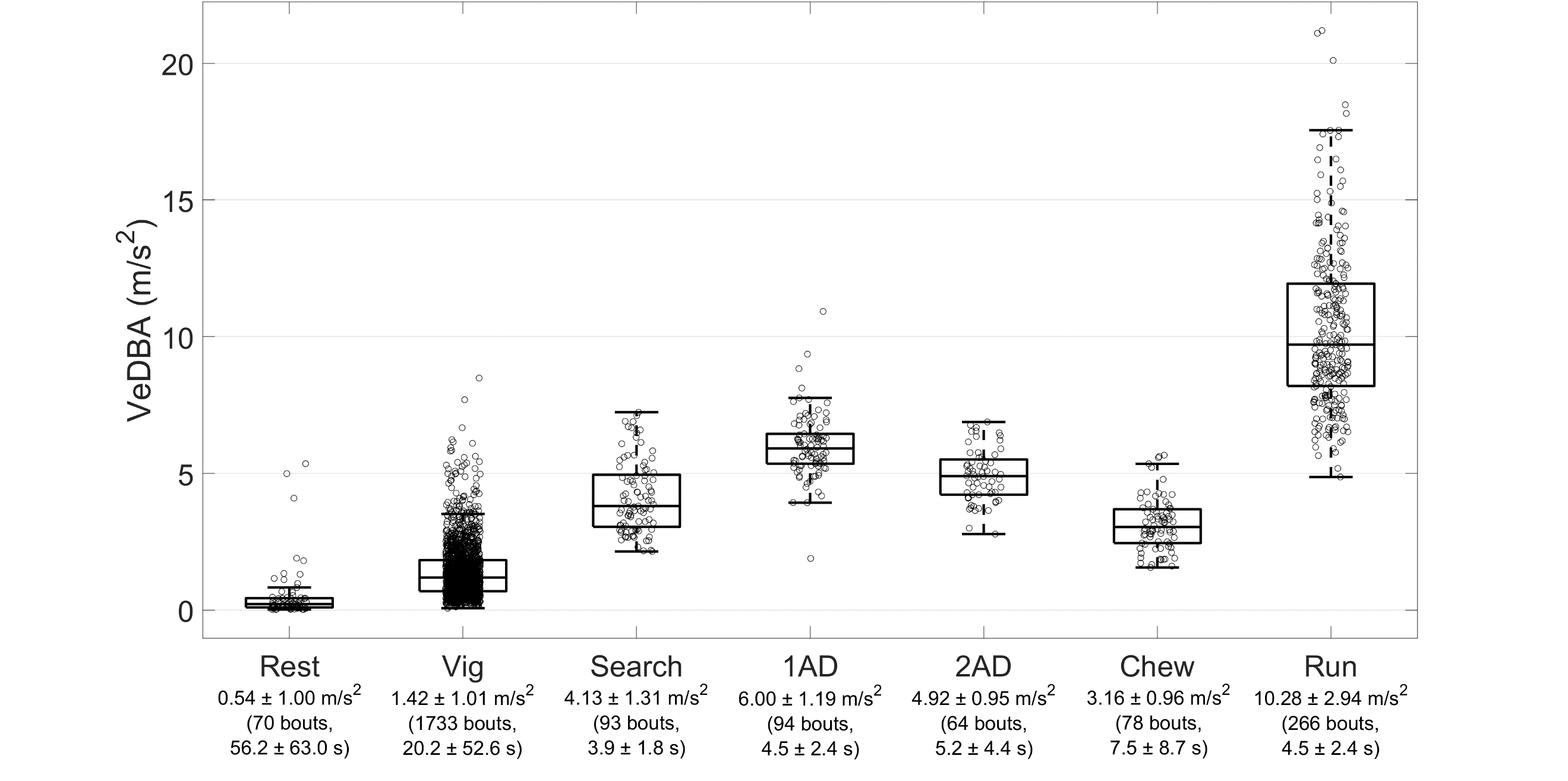


Figure S1. **Behaviour-specific vectorial dynamic body acceleration (VeDBA) for meerkats**. We calculated behaviour-specific VeDBA from acceleration data in the power calibration dataset. Roughly speaking, there are three ‘levels’ of VeDBA values: low (resting, vigilance), medium (searching, 1AD, 2AD, chewing), and high (running). Reported here are standard mean and deviation of VeDBA across bouts, and number of bouts with mean and s.d. of bout duration (in seconds). Vig: vigilance; 1AD: one-armed digging; 2AD: two-armed digging.

#### References

Chakravarty, P., Cozzi, G., Ozgul, A., & Aminian, K. (2019). A novel biomechanical approach for animal behaviour recognition using accelerometers. *Methods in Ecology and Evolution*, *10*(6), 802-814.

Scantlebury, M., Russell, A. F., McIlrath, G. M., Speakman, J. R., & Clutton-Brock, T. H. (2002). The energetics of lactation in cooperatively breeding meerkats Suricata suricatta. *Proceedings of the Royal Society of London. Series B: Biological Sciences*, *269*(1505), 2147-2153.

Scantlebury, M., Clutton-Brock, T. H., & Speakman, J. R. (2004). Energetics of cooperative breeding in meerkats Suricata suricatta. In *International Congress Series* (Vol. 1275, pp. 367-374). Elsevier.
